# Failures in Clinical Trials in the European Union: Lessons from the Polish Experience

**DOI:** 10.1007/s11948-012-9400-9

**Published:** 2012-10-06

**Authors:** Marcin Waligora

**Affiliations:** Department of Philosophy and Bioethics, Jagiellonian University, Medical College, ul. Michalowskiego 12, 31-126 Krakow, Poland

**Keywords:** RECs, Clinical trials, Biomedical research, Poland, Central and Eastern Europe, Failures

## Abstract

When discussing the safety of research subjects, including their exploitation and vulnerability as well as failures in clinical research, recent commentators have focused mostly on countries with low or middle-income economies. High-income countries are seen as relatively safe and well-regulated. This article presents irregularities in clinical trials in an EU member state, Poland, which were revealed by the Supreme Audit Office of Poland (the NIK). Despite adopting many European Union regulations, including European Commission directives concerning Good Clinical Practice, these irregularities occurred. Causes as well as potential solutions to make clinical trials more ethical and safer are discussed.

## Introduction

In contemporary debates about research ethics, most attention is given to countries with relatively weak regulations in this field. When talking about safety of research subjects, exploitation, vulnerability and irregularities in clinical research, commentators focus mostly on the developing countries with low or middle income economies (Benatar 2004; Benatar and Fleischer 2007; Emanuel 2008; Emanuel et al. 2004; Hyder et al. 2009; Levine 1999; Macklin 2004). High-income countries are seen as relatively safe and well-regulated.

This article discusses failures, irregularities and malpractice in clinical trials in a European Union member state, Poland. I believe that Poland is interesting for at least two reasons:

First, Poland had to adopt many European Union legal regulations, including two directives concerning Good Clinical Practice (Directive 2001/20/EC, Directive 2005/28/EC). Moreover, the number of clinical trials per year carried out in Poland is relatively high: about 30000 human subjects participate in clinical trials yearly. About 450 new clinical trials of medical products are registered annually (Report 1, 2010). Poland is ranked 10th in the world and 1st among emerging markets in terms of number of clinical trial sites (PricewaterhouseCoopers 2010).

Second, Poland is a relatively new EU member state (2004). A few years ago it was also classified by The World Bank as a high-income country (The World Bank web page). However, it is still perceived as an emerging market and transition society. Therefore, Poland is a good example of the position of a new member state as well as an example of possible future circumstances in countries still under development. Lessons from Poland can benefit others.

This paper focuses on two reports published in 2010 by the Supreme Audit Office of Poland (Najwyższa Izba Kontroli or the NIK) based on two audits. The first audit was devoted to, among other items, the inspection of external sources of financial support, including clinical research external funds. A result of an audit report was published in July 2010. The second was devoted to an audit of supervision quality of clinical hospitals and trials conducted in 13 clinical hospitals. That report was published in December 2010. In both reports, many irregularities and errors were described in the conduct of clinical research.

## Auditors and Reports

Audits were performed by the Supreme Audit Office of Poland. It is the supreme body of audit subordinate to the lower chamber of the Polish Parliament, acting in accordance with the principle of collegiate responsibility. The NIK controls the execution of the state budget, as well as the quality of management and potential irregularities in public institutions. The NIK undertakes audits ordered by the lower chamber of Parliament, the Prime Minister, the President, or on its own initiative. The NIK is also a member of The International Organization of Supreme Audit Institutions (INTOSAI) as well as the European Organization of Supreme Audit Institutions (EUROSAI). The Supreme Audit Office of Poland is a politically independent body, e.g. The NIK President is protected by immunity, and is appointed by the lower chamber of Parliament for a 6 years term of office; the term of office of Parliament is 4 years. With decades of activities, the Supreme Audit Office of Poland has achieved a very high reputation.

Audits undertaken by the NIK are based on Polish legal regulations, The International Organization of Supreme Audit Institutions Auditing Standards, the European Implementing Guidelines for the INTOSAI Auditing Standards, the Standards of the International Federation of Accountants (IFAC) as well as on the long experience of the NIK (http://www.nik.gov.pl/en/nik-audits/standards/).

The NIK is one of two institutions able to perform an extensive external audit of biomedical research. It should be stressed however, that the NIK is not competent body to audit medical and methodological issues of biomedical research. The second (and the main one) body able to perform an external audit is The Office for Registration of Medicinal Products, Medical Devices and Biocidal Products.

The first audit (Report 1, 2010) was initiated by the NIK after alarming results indicated lax controls in a Katowice hospital in 2007. The audit was planned and previously announced by the NIK. It included inspecting external sources of financial support, including clinical research external funds. The audit was performed between December 2008 and June 2009, focusing on the years 2006–2008. A report was published in July 2010. Thirteen clinical hospitals associated with medical schools were controlled at this time. The audit encompassed at least 1959 clinical trials carried out in all controlled hospitals. Results of this audit were quite alarming.

The second audit (Report 2, 2010) was performed because of results that caused concern and many failures and irregularities found by the NIK during the first audit. The NIK did not audit the scientific validity of clinical trials performed in controlled hospitals. The second audit focused on the quality of supervision of clinical hospitals and trials. It was conducted in 13 medical universities in Poland (which are obliged to supervise university hospitals) by The Office for Registration of Medicinal Products, Medical Devices and Biocidal Products as well as the Ministry of Health. The NIK focused also on the audit agreements between medical universities and sponsors of clinical trials as well as the functioning of Ethics Committees (RECs) established by medical universities. The audit was conducted between December 2009 and May 2010, focusing on the years 2006–2009. A report was published in December 2010.

## The Structure of Bodies Involved in Supervision, Organization and Conduct of Clinical Trials

**Fig. 1:**
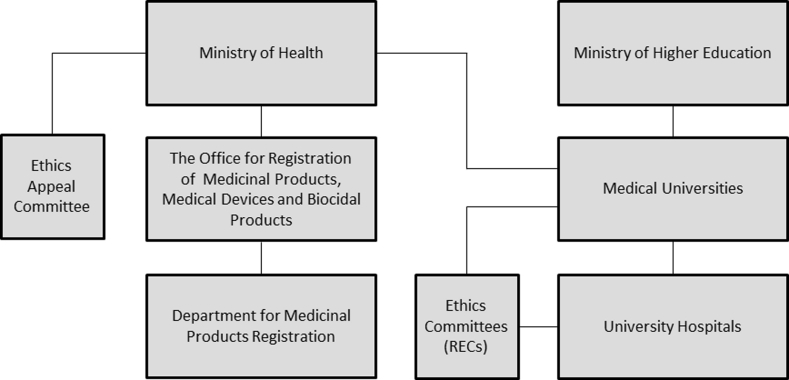
The structure of the bodies described in reports published by the Supreme Audit Office of Poland (the NIK)

The main requirement to conduct clinical trials in Poland is to obtain approval by the Bioethics Committee (Polish equivalent of Research Ethics Committees or RECs) as well as permission by The Office for Registration of Medicinal Products, Medical Devices and Biocidal Products. The President of the Office is a government administrative authority responsible for, among others, marketing authorization of medicinal and biocidal products, medical devices as well as, within a limited scope, clinical trials. The Office is a public administration body supporting the President of the Office in the realization of those matters. One of the units of the Office for Registration is a Department for Medicinal Products Registration. The President of the Office for Registration is elected by the government and is subordinate to the Ministry of Health (Fig. 1).

Research Ethics Committees are independent bodies. They are created at the medical universities (appointed by the Chancellor), regional chambers of physicians and dentists (appointed by the Regional Council of Physicians and Dentists) and medical research centers (appointed by the Head of the Center) (Act on professions of physicians and dentists 1996). Medical research projects which have been negatively assessed by the REC could be sent to the Ethics Appeal Committee (appointed by the Ministry of Health). Czarkowski and Różanowski emphasized that RECs’ work is not coordinated and there is no central institution that could monitor its work (Czarkowski and Różanowski 2009).

Medical universities are independent bodies, however financed mostly from the public funds. Medical universities have the right to establish university hospitals. University hospitals are governed by the head of the hospital, by the board and supervised by the chancellor of the medical university. Medical universities apply for the contracts and funds for covering the cost of certain medical procedures to the National Health Fund (the NFZ). This is the main source of their income. The National Health Fund is an authority responsible for, among others, planning and purchasing publicly financed health services. Expenses of the National Health Fund are covered by the mandatory social health insurance contributions. The National Health Fund is managed by the President, who is appointed by the National Health Fund Council. The Council is appointed by the Prime Minister (Kuszewski and Gericke 2005).

## Findings of the Supreme Audit Office of Poland (the NIK) Audits

In the conclusion of the first report, the authors wrote: „In the field of clinical trials the NIK found uneconomical, illegal and unreliable activities as well as the potential to expose patients to harm […] Pharmaceutical companies […] have more influence in conducting, financing and financial control of clinical research than public institutions” (Report 1, 2010). The findings of the audits published in these reports are categorized below in terms of (1) failures of the national level bodies to fulfill their oversight and management responsibilities, (2) failures of regional and local institutions. Such categorization facilitates analysis.

1. Failures of national level institutions
  - The NIK judged unfavorably „a lack of a comprehensive system of regulations concerning conducting, financing and financial control of clinical research”.
  - The NIK criticized the unreliable supervision of the Ministry of Health over The Office for Registration of Medicinal Products. The lack of clear and detailed legal regulations devoted to functioning of the Central Register of Clinical Trials (the Department of The Office for Registration of Medicinal Products) was the main reason for the lack of reliable verification of related documentation (Report 2, 2010).
  - “The Office for Registration of Medicinal Products gave permission for research conducted without reliable verification of related documentation” (Report 2, 2010).
  - The most alarming examples are (1) A case of research approval given by The Office for Registration of Medicinal Products, ignoring the fact that the protocol for this research had been previously disapproved by the REC (Report 2, 2010). (2) A case of research approval given by The Office for Registration of Medicinal Products without the REC’s approval (Report 2, 2010). (3) A case of research approval given by The Office for Registration of Medicinal Products for substantial changes in ongoing research without the REC’s approval (Report 2, 2010).
  - The Department of The Office for Registration of Medicinal Products—Central Register of Clinical Trials did not collect and store full and valid information regarding registered clinical trials (e.g. data did not contain information regarding the number of subjects as well as basic information about research centers participating in the research). One of the reasons for this is a lack of detailed information describing a class of required data collected by the Central Register of Clinical Trials in Polish law. It should be noted that the Central Register of Clinical Trials is the only institution in Poland collecting detailed data regarding clinical trials (Report 2, 2010).
  - One of the most criticized irregularities was the lack of an appropriate number of audits undertaken by The Office for Registration of Medicinal Products, Medical Devices and Biocidal Products. The NIK considered that as one of the causes of many other irregularities. Only 1.3 % of the research projects were audited by this body (15 from 1130 registered trials). Moreover, only one audit was initiated by The Office; nine were initiated by the Food and Drug Administration (USA); three were initiated by the European Medicines Agency. The low number of audits was the main reason that the second audit devoted to the quality of supervision of clinical hospitals and trials was performed (Report 1, 2010).
2. Failures of regional and local institutions
  - Irregularities in 9 of 13 hospitals were identified as “possibly dangerous to life or health of research subjects” (Report 1, 2010).
  - Heads of all audited hospitals did not demand from sponsors’/investigators’ ethics committees approvals and also Ministry of Health decisions, as well as other documents required by law were not obtained. Therefore, heads of hospitals did not have reliable information regarding the number and types of clinical trials conducted in managed hospitals, as well as the number of subjects involved (Report 2, 2010).
  - Consequently, in two cases, trials started before the REC approval: The Clinical Evaluation on Advanced Resynchronization (CLEAR) and Multi-Center Autonomic Defibrillator Implantation Trial (MADIT) in Warsaw. In two cases, the NIK found a lack of research protocols: Ongoing Telmisartan Alone and in Combination with Ramipril Global Endpoint Trial/Telmisartan Randomized Assessment Study in ACE Intolerant Subjects with Cardiovascular Disease (ONTARGET-TRANSCEND) and A Prospective Multicentre Registry to Evaluate the Efficacy and Safety of Treating Patients with De Novo Coronary after Lesions with a Paclitaxel-Eluting Coronary Stent System LUC-CHOPIN (LUC CHOPIN) in Katowice. Moreover, a defect was discovered in one of the pacemakers (Report 1, 2010).
  - Many procedures performed during the research were illegally classified as standard medical care and sponsored by the National Health Fund (the NFZ). In addition, purchase of some of the tested samples were performed by hospitals and paid for from public funds (the NFZ), (Report 1, 2010).
  - In many cases, the same physician/researcher conducted multiple research projects at the same time. In UCK Gdansk, one researcher conducted 75 research projects between 2006 and 2008 (Report 1, 2010).
  - None of the audited hospitals registered and counted their tested samples. Most of the samples were stored in inappropriate places, some of them after their expiration date.
  - The NIK unfavorably judged 11 of the 12 ethics committees (RECs) because „they violated rules regulating their functioning and financing” (Report 2, 2010). In the case of 6 committees, the NIK found that they approved research protocols despite lack of essential information and important documents (e.g. lack of agreements between researchers and research centers). In some cases, the REC’s members did not receive project approval before the final the REC meeting. In one instance, most of the REC’s approvals were signed only by the head of the REC, which made them invalid (Report 2, 2010).
  - Most of the medical universities did not fulfill the required conditions to act as a research center (requirements for such centers are included in legal regulations). Nevertheless, they undertook this role. In a few cases, the heads of the clinical hospitals did not know about research initiated in subordinate institutions (Report 2, 2010).

Most of the failures described were also violations of the principles of Good Clinical Practice contained in adopted Directives (Table 1).

**Table 1:** Articles of the Directives 2001/20/EC and 2005/28/EC violated by the irregularities

	Requirement	Irregularities
2001/20/EC (Directive 2001)		
Article 3, Sec. 2a	A clinical trial may be initiated only if the Ethics Committee and/or the competent authority comes to the conclusion that the anticipated therapeutic and public health benefits justify the risks and may be continued only if compliance with this requirement is permanently monitored	1. CLEAR and MADIT trials in Warsaw started before the REC approval 2. ONTARGET-TRANSCEND and LUC-CHOPIN trials in Katowice – the NIK found lack of research protocols
Article 6, Sec. 2	The Ethics Committee shall give its opinion, before a clinical trial commences, on any issue requested	As above
Article 9 Sec. 1	[…] The sponsor may not start a clinical trial until the Ethics Committee has issued a favorable opinion and inasmuch as the competent authority of the Member State concerned has not informed the sponsor of any grounds for non-acceptance	As above Research approval given by The Office for Registration of Medicinal Products, despite the fact that the protocol for this research had been previously disapproved by the REC
Directive 2005/28/EC		
Article 6 Sec 3	Communication of information between the Ethics Committees and the competent authorities of the Member States shall be ensured through appropriate and efficient systems	Lack of appropriate and efficient systems of communication between RECs and other units
Article 26	Member States shall establish the relevant procedures for verification of good clinical practice compliance The procedures shall include the modalities for examining both the study management procedures and the conditions under which clinical trials are planned, performed, monitored and recorded, as well as follow-up measures	Existing procedures seem to be insufficient The NIK found the lack of an appropriate number of audits undertaken by The Office for Registration of Medicinal Products, Medical Devices and Biocidal Products

## What Can Be Learned from the Supreme Audit Office of Poland reports? Analysis and Suggested Solutions

The Supreme Audit Office of Poland report shows that failures and irregularities in the field of clinical trials in Poland had various causes. Some of them were based on structural problems, such as: (1) lack of appropriate regulations, (2) lack of correct implementation of detailed procedures or (3) confusion between rules and definitions presented in national law and those presented in adopted Directives (Directive 2001/20/EC, Directive 2005/28/EC). The others are caused by conditions typical for transitional societies: (4) massive and rapid changes in modernized laws and rules, which are sometimes impossible to comprehend by the average citizen as well as by the leaders and administrative employees of modernized institutions, (5) habits of citizens of transitional societies not consistent with new rules, (6) the frequent phenomenon of low ethical standards (7) an aspiration to achieve the income and quality of life of developed societies which could badly impact research integrity. In the countries from the region of Central and Eastern Europe, additional factors are (8) a habit of opposition to institutions and laws of the previous historical (“Communist”) period and (9) a paternalistic approach among medical staff. Selected similar causes of the problems of research ethics systems were analyzed previously by Borovečki et al. (2005), Dranseika et al. (2011), Famenka (2011), Gefenas et al. (2010), Hyder et al. (2009), Silis (2010).

Selected identified failures 1–3 are analyzed below.

### Lack of appropriate regulations

In general, the legal situation of regulated clinical trials in Poland is quite satisfactory. It should be stressed that there is no alarming lack of appropriate law. The most important factors influencing legal improvement were two Directives (Directive 2001/20/EC, Directive 2005/28/EC) implemented into existing law.

The poorest regulated area is the field of conflict of interest in biomedical research. The phenomenon of conflict of interest is barely identified. This is not a typical problem of Polish law only, but rather of the entire Central and Eastern Europe Region (Górski 2002; Gefenas et al. 2010; Dranseika et al. 2011). In Poland, conflict of interest in biomedical research is identified only in the *Physician’s Code of Ethics* (The Chamber of Physicians and Dentists, 2004). According to the *Code,* a physician-researcher is obliged, among others, to inform the patient-participant about the fact of the researcher’s connections with pharmaceutical companies as well as to disclose conflicts of interest in published articles and public presentations. Unfortunately, the legal status of the *Code* is not fully clear. It was legislated at the National Convention of Physicians and applies to members of that organization. However, the current version of the *Code* is criticized because of lack of consistency with binding national law.

The second weakly regulated area, according the Supreme Audit Office, is the field of regulation concerning financial control of clinical trials. Here new regulations are definitely needed.

Suggestions for improvement:

- *Central institutions* It is suggested that a legal solution be introduced regarding conflict of interest in biomedical research. It is suggested that comprehensive regulations are implemented concerning financial control of clinical research. A new bill, *Law of clinical trials with clinical therapeutic products and veterinary therapeutic products,* regulates financial control appropriately. This bill will complement many other legal solutions suggested by the Supreme Audit Office reports. The current version of this bill was published by the Ministry of Health on its web site in 2011 (Ministry of Health 2011). To make it valid as a binding law, the bill must be passed by the Polish Parliament and signed by the President of Republic of Poland.
- *Regional bodies* It is strongly recommended the introduction of a conflict of interest policy in the field of clinical research at the medical universities and hospitals. An example of that policy could be the revised Harvard University Policy of Individual Financial Conflict of Interest for Persons Holding Faculty and Teaching Appointments approved in 2010 (Harvard University COI Policy 2010). Unfortunately, no action was taken on this matter in Poland after the Supreme Audit Office reports.

### Lack of correct implementation of detailed procedures

Implementation of Directives regarding Good Clinical Practice in Poland was not preceded by detailed legal solutions. That was one of the reasons for many irregularities and failures. The Supreme Audit Office criticized gaps in detailed regulations devoted to the functioning of the Central Register of Clinical Trials as well as lack of regulations describing detailed information about types of required data collected by the Central Register of Clinical Trials.

Suggestions for improvement:

- *Central institutions* Most of the gaps in the regulation’s detailed procedures were already filled by new legislation following the Supreme Audit Office reports: *Act of the Office for Registration of Medicinal Products, Medical Devices and Biocidal Products* (Act on the Office for Registration 2011), *Order of the Ministry of Health regarding inspection of clinical research* (Ministry of Health 2012b). However, the most important, the new bill, *Law of clinical trials with clinical therapeutic products and veterinary therapeutic products* discussed above, has not yet been enacted into law Unfortunately, it does not contain regulations regarding the supervision of the REC’s activity, which is definitely required (Czarkowski and Różanowski 2009).
- *Regional bodies* In reaction to the Supreme Audit Office reports, Jagiellonian University Medical College established a new institution: The Unit for Clinical Research. This body is responsible for constant supervision and monitoring of clinical trials conducted at the Medial College as well as administrative support of the trials. The Unit for clinical research worked out Standard Operating Procedures (SOPs) regarding the conduct of clinical trials at the Medical College. It is suggested that other medical universities establish similar institutions.

### Confusions between rules and definitions presented in national law and those presented in adopted Directives (Directive 2001/20/EC, Directive 2005/28/EC)

Adaptation of Directives concerning Good Clinical Practice (Directive 2001/20/EC, Directive 2005/28/EC) were introduced into Polish law by amendment to the Pharmaceutical Law as well as by the orders of the Ministry of Health (Ministry of Health 2005, 2012a). The new rules were mixed with the old rules and definitions. The result is highly unsatisfactory. The current version of Pharmaceutical Law is unclear, hard to understand and chaotic. Moreover, there are currently several regulations that govern clinical trials in Poland. Definitions such as “patient” and “physician” (instead of “participant” and “researcher”) “medical experiment”, “therapeutic experiment” and “non-therapeutic experiment” are still used in Polish law despite the fact that they were criticized harshly (Levine 1999). A lack of clarity is definitely one of the reasons for so many irregularities in the clinical trials. Furthermore, the outdated and often harshly criticized Polish *Physician’s Code of Ethics* is not consistent with existing national law in the matter of clinical experiments. For instance, Polish law does not allow the conduct of clinical trials with soldiers as participants, while the *Code* does allow this practice (The Chamber of Physicians and Dentists 2004).

Suggestions for improvement: It is suggested that key definitions are improved especially those connected to biomedical research used in Polish law and to make them coherent, clear and consistent with international documents. Some of the improvements are included in the current version of the new bill, *Law of clinical trials with clinical therapeutic products and veterinary therapeutic products*. It is suggested that this be passed as soon as possible. It is also suggested that the National Convention of Physicians update the *Physician’s Code of Ethics* to make it consistent with Polish and international laws.

## Conclusion

Failures in organizing, conducting and supervising clinical trials in Poland showed that more attention should be given to biomedical research in countries with regulations which seem to be appropriate. As an European Union member state, Poland was required to adopt European Commission directives concerning Good Clinical Practice. Unfortunately, it was not enough to stop all the irregularities. It was demonstrated that failures could have many reasons: some are typical for transition societies and emerging markets, the other mostly for so called post-Communist countries. A positive side of disclosing failures described by the Supreme Audit Office of Poland is a strong impact for legal and organizational improvements concerning supervised areas. That lesson can benefit others, especially, when all the improvements which are already started will continue and be fully implemented.
